# Heart Rate and Cardiovascular Disease: An Alternative to Beta Blockers

**DOI:** 10.4061/2009/179350

**Published:** 2009-07-30

**Authors:** Michael Liang, Aniket Puri, Gerard Devlin

**Affiliations:** Department of Cardiology, Waikato Hospital, Pembroke & Selwyn Sts, Private Bag 3200, Hamilton 3240, New Zealand

## Abstract

Ivabradine, an *I*_*f*_ inhibitor, acts primarily on the sinoatrial node and is used to reduce the heart rate with minimal effect on myocardial contractility, blood pressure, and intracardiac conduction. Heart rate reduction is an important aspect of care in patients with chronic stable angina and heart failure. Many patients with coronary artery disease have coexisting asthma or chronic obstructive airway disease, and most of them are unable to tolerate beta blockers. Ivabradine may thus be a useful medicine in therapeutic heart rate management especially in patients who are intolerant of beta-blockers.

## 1. Introduction

A higher heart rate is associated with an increase in cardiovascular mortality both in general population and in patients with established cardiovascular disease [1–4]. Previous studies have shown that a rapid heart rate is a risk factor for developing hypertension and atheroscelerosis [5–7]. In patients with suspected and established coronary artery disease, an elevated heart rate is an independent predictor of survival [8–11]. The morbidity-mortality evaluation of the *I*_*f*_ inhibitor ivabradine trial (BEAUTIFUL trial) showed that a heart rate greater than 70 beats per minute (bpm) is associated with increased cardiovascular death [11]. 

 Lower heart rate reduces myocardial oxygen consumption and increases oxygen supply to heart via prolongation of diastole [12]. This property is important in patients with chronic stable angina. The stenotic coronary arteries often receive oxygen supply from less severely stenotic arteries via collateral branches. Blood flow in collateral branches is optimized in prolonged diastolic phase. An increase in heart rate results in a shortened diastole and therefore reduces blood supply to the stenotic coronaries via collaterals. Maintaining a stable lower heart rate in these conditions is beneficial for symptom control [13]. 

 Persistent elevated heart rate is also commonly seen in patients with congestive heart failure (CHF). Studies in the past using drugs either increasing or reducing heart rate showed survival benefit when treatment group had lower average heart rate than placebo. By contrast, survival was poorer when average heart rate of the treated cohort had increased compared with placebo [14]. 

In addition to existing beta-blockers and non-dihydropyridine calcium channel inhibitors, *I*_*f*_ inhibitors that blocks the *I*_*f*_ current in sinoatrial node may have a role in therapeutic heart rate management and angina control [12]. This could be particularly useful in patients who are unable to tolerate beta-blockers or non-dihydropyridine calcium channel inhibitors. In New Zealand, the prevalence of asthma is nearly 20% and it is common to come across this group of patients who are unable to tolerate beta-blockers [15]. The concept and application of *I*_*f*_ inhibitor will be reviewed in this article.

## 2. The Concept of *I*_*f*_ Inhibition

**Figure 1:**
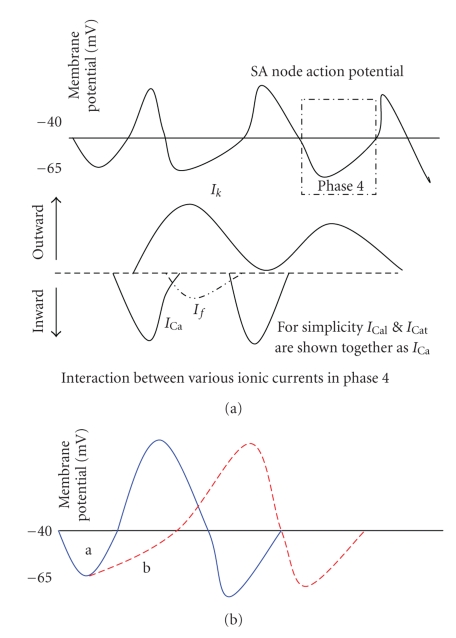
(a) a diagram demonstrates I_*f*_ current and other ionic currents in phase 4 of action potential. For simplicity *I*_caL_ and *I*_caT_ are shown together as *I*_ca_. (b) Inhibition of *I*_*f*_ phase 4 of action potential leads to reduction of slope from a to b. This results in prolonged phase 4 or firing frequency of action potential, thus slower heart rate.

*I*_*f*_ current, first described by Brown et al. in 1979, is activated in phase 4 of action potential in the sinoatrial node by accelerating diastolic depolarisation [16]. Phase 4 of the cardiac action potential is the resting membrane potential and a cell remains in this phase until it is triggered by external electrical stimulus. The sinoatrial node demonstrates automaticity by spontaneous depolarisation without external electrical or nervous stimulus. This is mediated by HCN channels (Hyperpolarization-activated, Cyclic Nucleotide-gated channels) which allows net inward mixed Na^+^/K^+^ current during diastole, that is, phase 4 action potential [13]. Because of this unusual behaviour it is named “funny” current and therefore so-called pacemaker current (*I* for current, *f* for funny) [17]. Three other ionic currents associated with this phase of action potential are *I*_k_, *I*_caL_, *I*_caT_. *I*_k_ is activated by preceding action potential and results in outward movement of potassium through the slow delayed rectifier potassium channels. The two inward calcium currents are *I*_caL_ (L type/long-lasting Ca channel) and *I*_caT_ (T type/transient Ca channel). The action potential reaches its threshold (approx. −40 mv) by *I*_*f*_ current which in turn controls the successive action potential and heart rate (see Figures 1(a)  and 1(b)).

The hyperpolarization-activated, cyclic nucleotide-gated (HCN) channels are responsible for *I*_*f*_ current. There are four isoforms of HCN channels that is, HCN1–HCN4 which can be found in heart, brain and retina. The main isoform found in the heart is HCN4 and it is highly expressed in sinoatrial node [18]. HCN4 channels are also present in atrio-ventricular node and Purkinje fibres; however, these are not active under normal physiological condition. *I*_*f*_ current might be involved in the pathological role of congestive heart failure and ventricular hypertrophy, therefore, it is attractive to search for potential pharmacological inhibitors which could result in heart rate reduction with minimal side effects.

## 3. Ivabradine—the Selective and Specific *I*_*f*_ Inhibitor

Few agents were developed for *I*_*f*_ inhibition in the past; the first of which is Alinidine, a clonidine derivative, that was soon abandoned due to its relative inotropic action [19]. Later, zetabradine, a benzazepinone derivative also went out of contention due to unacceptable ocular sideeffects and QTc prolongation [20, 21]. 

 Ivabradine, a unique specific *I*_*f*_ current inhibitor, was first described by Thollon et al. more than a decade ago [22]. It exhibit dose-dependent heart rate reduction with minimal effect on myocardial contractility, blood pressure, intracardiac conduction and ventricular repolarisation [23]. At the treatment dose, ivabradine has no effect on electrocardiographic PR or QT (QTc) interval. When compared with beta-blocker atenolol, ivabradine depresses myocardial relaxation to a lesser extent both at rest and exercise [24, 25].

## 4. Adverse Effects with Ivabradine

Common side effects from Ivabradine resulting in withdrawal of treatment are; visual symptoms such as blurred vision, phosphenes and visual disturbance. Clinical trials suggest dose-dependent reversible visual side effects are relatively small in treatment dose up to 10 mg twice daily [23, 26, 27]. 

 In the BEAUTIFUL trial, symptomatic bradycardia was the most common adverse effect leading to discontinuation of treatment, however, 87% of enrolled patients were receiving beta-blockers at the same time [23]. 

 Since ivabradine targets sino-atrial node for heart rate control, its use was discouraged in the presence of atrial fibrillation, persistent pacemaker rhythms and second/third degree atrio-ventricular block [11, 22, 26].

## 5. Clinical Trials of Ivabradine

The international BEAUTIFUL trial is, to date, the largest randomized, double-blinded, placebo-controlled trial of ivabradine [11, 23]. Patients with coronary artery disease and left-ventricular ejection less than 40% were included in this trial. Out of 10917 eligible patients, 5479 patients received 5 mg ivabradine gradually titrated to the target dose of 7.5 mg twice a day in addition to conventional cardiovascular medication. Patients receiving ivabradine had 6 bpm reduction in heart rate from the mean baseline of 71.6 bpm at 12 months. However, 87% of patients were also on a beta-blocker. The trial showed that there is no difference in primary composite outcome, cardiovascular death or admission to hospital for new-onset or worsening heart failure in the study population. However, the analysis of prespecified subgroup of patients with a heart rate of 70 bpm or greater shows a lower rate of admission to hospital for myocardial infarction (HR 0.64, *P* = .001), myocardial infarction or unstable angina (HR 0.78, *P* = .23), and coronary revascularisation (HR 0.70, *P* = .16). These findings suggest a heart rate of 70 or greater in the presence of coronary artery disease and moderate systolic failure is associated with increased cardiovascular death, hospital admission for heart failure or myocardial infarction and coronary revascularisation. The study also implies that a heart rate of 75 or less could be target for therapeutic heart rate management in this group of patients who have higher baseline heart rate. 

 The efficacy of ivabradine as monotherapy in angina control was tested in a randomized double-blinded trial by Borer et al. [28] 360 patients with chronic stable angina were assigned to either placebo or ivabradine (2.5, 5 or 10 mg twice daily) as the antianginal monotherapy plus short-acting nitrates for 2 weeks. Prior to the randomization, the eligible patients had an initial 2–7 days of antianginal medication washing out; this included beta-blockers, calcium channel blockers and long-acting nitrates. Bicycle ergometric exercise tests were performed at the initial inclusion and end of 2 weeks to assess time to 1 mm horizontal ST depression and limiting angina. After 2 weeks, the study was converted to an open-label 2 or 3-months extension on ivabradine 10 mg twice daily and then a 1-week randomized withdrawal to ivabradine 10 mg twice daily or placebo. At the end of 1-week, another exercise test was performed. 

 Of the 360 eligible patients, 103 were excluded due to protocol violation and the majority of them had negative exercise tests at the time of randomization. In the remaining 257 patients, the time to horizontal ST segment depression of ≥1 mm, onset of angina and limiting angina is longer in all ivabradine dose groups compared with the placebo, reaching a statistical significance (*P* = .04) at the dose of 10 mg twice daily. Resting and exercise heart rate in ivabradine groups are significantly lower than placebo (*P* < .05). In addition, the dose-dependent response was observed. The frequency of angina attacks and use of short-acting nitrates were also decreased in patients into the open-label extension (*P* = .001). During the randomized withdrawal, the frequency of angina increased in placebo group but remained unchanged in ivabradine group. 

 The (International Trial of the Antianginal effects if Ivabradine Compared to Atenolol) INITIATE study involved 939 patients with stable angina randomized into ivabradine 5 mg bid for 4 weeks followed by either 7.5 or 10 mg bid for 12 weeks or atenolol 50 mg od for 4 weeks then 100 mg od for 12 weeks [26]. All patients underwent exercise stress tests at the time of randomization and after 4 and 16 weeks of therapy. Total exercise duration at the end of the 4 weeks did not show significant difference in ivabradine and atenolol groups. The number of angina attacks was decreased by two-thirds with both ivabradine and atenolol. The study concluded that ivabradine is as effective as atenolol in patients with stable angina. 

 The Antianginal property of ivabradine was also compared with amlodipine (dihydropyridine calcium channel blockers) in a 3 months double-blind trial [29]. 1195 patients with a ±3-month history of chronic stable angina and documented CAD were randomized to three groups—ivabradine 7.5 mg bid (*n* = 400), ivabradine 10 mg bid (*n* = 391) and amlodipine 10 mg od (*n* = 404). Patients underwent bicycle exercise tolerance tests at randomisation and every month for three months. The exercise tolerance, time to limiting angina, time to angina onset and time to 1 mm ST segment depression were consistently increased in all groups without significant difference. Of interest, heart rates at rest and exercise were significantly lower in ivabradine groups compared with amlodipine. 

 Combination of ivabradine and dihydropyridine calcium channel blockers was noted to be safe and well tolerated in another trial which allowed concomitant medications including long-acting nitrates or dihydropyridine calcium channel blockers [30]. The resting heart rate was reduced by 10 bpm in patients receiving ivabradine 5 mg bid and 12 bpm in patients receiving 7.5 mg bid. Of the 386 patients, 4 patients withdrew treatment due to visual side effects and 3 patients had sinus bradycardia requiring discontinuation of ivabradine. No ECG abnormality was detected in this study. The number of angina attacks per week showed a significant reduction at the end of 1 year study in both ivabradine 5 mg bid and 7.5 mg bid groups.

## 6. Conclusion

Available knowledge to date indicates that heart rate over 70 bpm in patients with coronary artery disease and systolic heart failure of ejection fraction of less than 40% is associated with adverse cardiac events. Currently, the optimum therapeutic heart rate in these groups of patients remains uncertain. The selective *I*_*f*_ inhibitor, ivabradine, provides an alternative way of heart rate reduction in addition to beta-blockers and calcium channel blockers. This could become particularly useful in patients who are intolerant of beta-blockers, for example, in the presence of asthma or severe chronic obstructive airway disease. At the treatment dose, it reduces heart rate to a similar extent as atenolol and can be used together with beta-blockers. Ivabradine is well tolerated in all the clinical trials with minimal side effects. The most common adverse effect is reversible visual symptoms. However, the absence of sinus rhythm is currently the contraindication of its use. 

 Further research is required to evaluate the mortality benefit of ivabradine in patients with known coronary artery disease and systolic heart failure who are unable to take beta-blockers. Clinical data suggests that ivabradine can be used as an antianginal medication as monotherapy or in combination with beta-blockers or calcium channel blockers. Its Antianginal effects are comparable to atenolol and amlodipine in clinical trials. Selective *I*_*f*_ inhibitor is likely to become another useful group of medicine in cardiovascular disease management in the near future.
